# Episodes of aggression and psychomotor agitation in psychiatric inpatients during the period of Covid-19 pandemia

**DOI:** 10.1192/j.eurpsy.2023.1828

**Published:** 2023-07-19

**Authors:** F. Franza, B. Solomita

**Affiliations:** 1 Psychiatry, Psychiatric Rehabilitation Centre Villa dei Pini, Avellino, Italy

## Abstract

**Introduction:**

The COVID-19 pandemic has changed social, family, and interpersonal relationships and behaviors. Several studies have identified the increase in psychiatric disorders in the general population (Fountoulakis *et al.* Psychiatry Res 2022; 315 114702) and an increase in episodes of disease in people already affected by these diseases (Taquet *et al.* Lancet Psychiatry 2022; 9 815-827; Zhu *et al.* Adults Psychiatry Res 2021; 301 113959). These episodes were accentuated by the severe limitations that occurred during the greatest peaks of the pandemic. However, few studies have evaluated the effects of these restriction periods on the levels of hetero or self-directed aggression in patients staying in residential facilities.

**Objectives:**

To evaluate the effects of the COVID-19 pandemic on aggression and psychomotor agitation crises in patients hospitalized in a psychiatric rehabilitation centre.Evaluate the differences in these behaviors in the different stages of external limitation in patients suffering from psychiatric disorders.

**Methods:**

This observational study was conducted in a residential psychiatric rehabilitation facility since the outbreak of the COVID-19 pandemic in Italy (March 2020 through September 2020). 354 patients were enrolled in the study. All guests had psychiatric disorders defined according to DSM-5 diagnostic criteria. Table 1 shows age mean and diagnosis at admission.

The several periods were recorded in the number of cases of psychomotor agitation and heterodirect aggression and an increase in emergency pharmacological interventions.

The following rating scales were administered in all patients: BPRS-18, BPRS Agitation (item item 6 tension + 10 hostility+ 17 excitement), GAF, and Epitrack.

The collected data were collected and statistically analyzed with the EZAanalyze 3.0 software in the Microsoft Excel Office.

**Results:**

In tables 1 and 2 and in Graphics 1 and 2 the results obtained from our study are shown. We observed a reduction in the number of agitation and aggression episodes in periods 1, 3 and 6 (4, 12 and 5, respectively). In these same periods, the ***BPRS agit*** subscore score was also lower than in the other periods (7.92, 8.08, 7.42, respectively).

**Image:**

**Figure:**
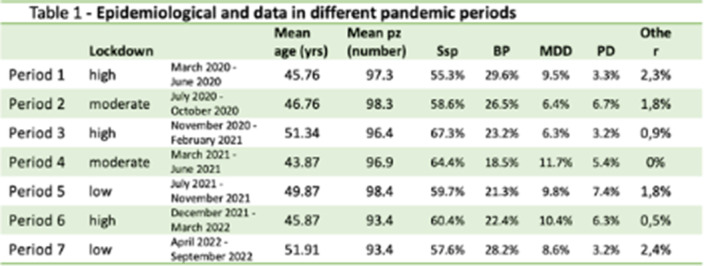

**Image 2:**

**Figure:**
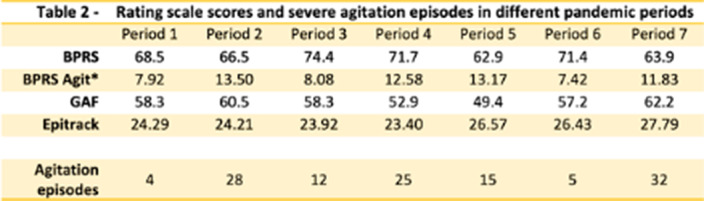

**Image 3:**

**Figure:**
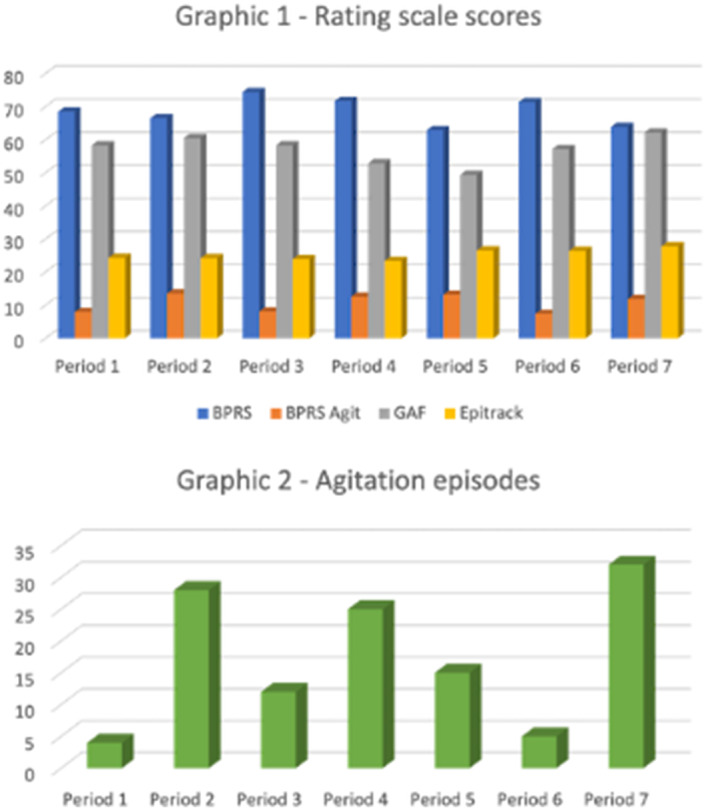

**Conclusions:**

Differently to expected data, the number of episodes of psychomotor agitation and heterodirect aggression proved to be reduced in the periods of greater restraint and limitation. Instead, the levels increased in the month following the reduction of such restrictions characterized by visits with family members, planned outings. it is difficult to give a reliable and definitive explanation to these results. However, the feeling of protection, risk reduction and potentially dangerous stimuli could guide the explanation of the results obtained.

**Disclosure of Interest:**

None Declared

